# Temperature Responsive Polymer Conjugate Prepared by “Grafting from” Proteins toward the Adsorption and Removal of Uremic Toxin

**DOI:** 10.3390/molecules27031051

**Published:** 2022-02-03

**Authors:** Erika Yoshihara, Makoto Sasaki, Ahmed Nabil, Michihiro Iijima, Mitsuhiro Ebara

**Affiliations:** 1 Research Center for Functional Materials (RCFM), National Institute for Materials Science (NIMS), 1-1 Namiki, Tsukuba 305-0044, Japan; erika.0530.y@gmail.com (E.Y.); s2020340@s.tsukuba.ac.jp (M.S.); DRNABIL_100@hotmail.com (A.N.); 2 Graduate School of Pure and Applied Sciences, University of Tsukuba, 1-1-1 Tennodai, Tsukuba 305-8577, Japan; 3 Biotechnology and Life Sciences Department, Faculty of Postgraduate Studies for Advanced Sciences (PSAS), Beni-Suef University, Beni-Suef 62511, Egypt; 4 Egyptian Liver Research Institute and Hospital (ELRIAH), El Mansoura, 35511, Egypt; 5 Department of Materials Chemistry and Bioengineering, National Institute of Technology, Oyama College (NIT, Oyama College), 771 Nakakuki, Oyama 323-0806, Japan; iijima@oyama-ct.ac.jp; 6 Graduate School of Industrial Science and Technology, Tokyo University of Science, 1-3 Kagurazaka, Shinjuku, Tokyo 162-0825, Japan

**Keywords:** polymer-protein conjugates, poly(*N*-Isopropylacrylamide), serum albumin, grafting from, indoxyl sulfate

## Abstract

In this study, temperature-responsive polymer-protein conjugate was synthesized using a “grafting from” concept by introducing a chain transfer agent (CTA) into bovine serum albumin (BSA). The BSA-CTA was used as a starting point for poly(*N*-isopropylacrylamide) (PNIPAAm) through reversible addition-fragmentation chain transfer polymerization. The research investigations suggest that the thermally responsive behavior of PNIPAAm was controlled by the monomer ratio to CTA, as well as the amount of CTA introduced to BSA. The study further synthesized the human serum albumin (HSA)-PNIPAAm conjugate, taking the advantage that HSA can specifically adsorb indoxyl sulfate (IS) as a uremic toxin. The HSA-PNIPAAm conjugate could capture IS and decreased the concentration by about 40% by thermal precipitation. It was also revealed that the protein activity was not impaired by the conjugation with PNIPAAm. The proposed strategy is promising in not only removal of uremic toxins but also enrichment of biomarkers for early diagnostic applications.

## 1. Introduction

Polymer-protein conjugates (PPCs) have been extensively developed in the last decades both in academic and industrial areas. The synthesis of bioconjugate materials mainly utilizes natural amino acids that exist within protein and peptide structures. Introduction of polymers into protein makes it possible for new functionalities such as improving solubility, enhancing dispersibility and inhibiting proteolytic enzymes. Such approaches are known to have promising applications as therapeutic and/or diagnostic technologies [1]. Generally, there are two common strategies for introducing polymers into proteins in PPCs: “Grafting to” (GT) approach bonds pre-synthesized polymers to proteins with functional groups by reactive coupling. “Grafting from” (GF) approach, on the other hand, initiates site for polymerization in aqueous solution through functionalized protein [2].

Poly (*N*-isopropylacrylamide) (PNIPAAm) is one of the most extensively studied temperature-responsive polymers in PPCs, which has a lower critical solution temperature (LCST) of around 32 °C [3]. Based on this property, protein-PNIPAAm conjugates can possess a temperature-sensitive ON-OFF switching functionality and can be used for protein recovery by thermal precipitation around body temperature [4]. Mainly, the GT approach is used for the protein-PNIPAAm conjugates because of its high coupling reaction efficiency and variable reaction conditions. Hoffman et al. have successfully prepared conjugates with a series of amine-containing proteins including lysozyme, myoglobin, protein A, hemoglobin, albumin and γ-globulin by using NIPAAm copolymer with *N*-hydroxysuccinimide (NHS) [5,6]. They have also succeeded in simplifying the structure of protein-polymer conjugates by implementing radical polymerization of NIPAAm from the polymerization initiation site with a carbonyl group at the polymer ends, which allowed the protein to bind a single end attachment to the polymer chain [7,8,9]. Okano et al. have also synthesized semitelechelic PNIPAAm with NHS groups at the polymer ends and successfully conjugated it with antibodies [10,11]. The conjugation of primary amino group and activated esters has been also used to prepare responsive macromolecular bioconjugates through streptavidin rather than direct conjugation onto the target protein [12,13,14,15,16,17,18]. In addition, it has been recently reported the preparation of conjugates under biological conditions and the introduction of functional groups into proteins by modifying PNIPAAm-derived copolymers by click reactions [19,20,21,22,23]. However, these methods involve complex processes such as multistep synthesis of PNIPAAm-derived polymers, functionalization of the polymer end groups and associated purification. In addition, introducing polymers to protein is inefficient due to steric hindrance. To improve efficiency, there is need to add excess polymers, which is challenging due to lack of control to the numbers and positions of PNIPAAm introduced in the protein surface.

To overcome these drawbacks, GF approach has attracted considerable attention in recent years due to its one-step preparation procedure and simple purification. GF approach uses functionalized proteins as initiation sites from where monomers are polymerized through living radical polymerization in an aqueous solution. However, only few reports have investigated the introduction of PNIPAAm into proteins through reversible addition-fragmentation chain transfer (RAFT) polymerization [24,25,26,27,28]. These reports confirmed the successful living radical polymerization of NIPAAm from proteins and the retained activity of protein-polymer conjugates after PNIPAAm introduction. Other reports mention the conjugation of protein-PNIPAAm prepared through atom transfer radical polymerization (ATRP). However, the removal of copper catalyst as well as deoxidization of solvent have been big challenges for the biological applications [29].

**Figure 1:**
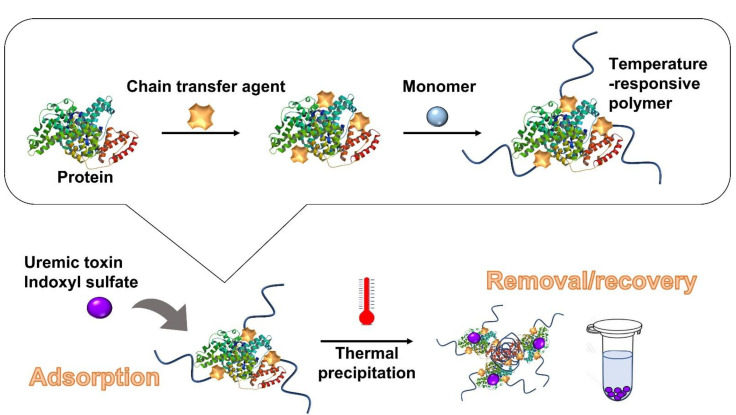
Schematic of the proposed thermally responsive polymer-protein conjugate system. First, protein-PNIPAAm was prepared by “grafting from” method. Next, the conjugate is allowed to capture indoxyl sulfate (IS), one of the uremic toxins. Finally, the captured IS is recovered by thermal precipitation.

**Scheme 1:**
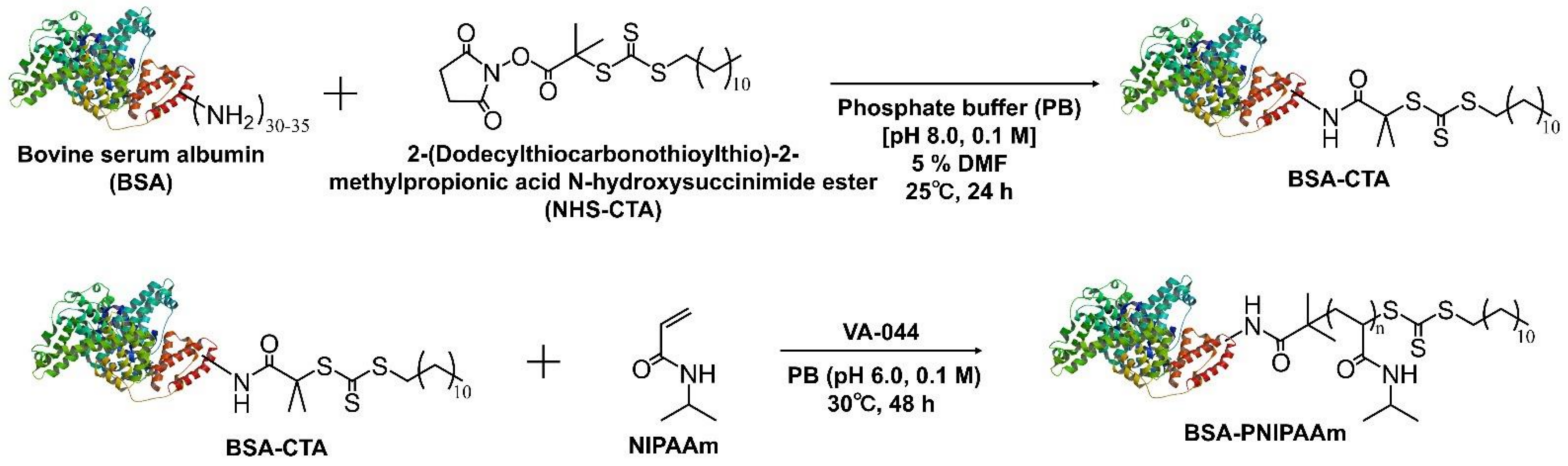
Synthesis of BSA-PNIPAAm conjugate by RAFT polymerization.

This study aims to prepare protein-PNIPAAm conjugates with the GF approach by functionalizing bovine serum albumin (BSA) with chain transfer agent (CTA) groups as an initiation site for the polymerization of NIPAAm (Scheme 1). Serum albumin was selected not only as a model protein, but also its adsorption ability for uremic toxin. The RAFT agent with NHS group (NHS-CTA) was used to react with the BSA amino group to synthesize macro-CTA. Moreover, in thermal RAFT polymerization, free linear polymers can be synthesized at the same time as the introduction of PNIPAAm into proteins and we believe that this will improve work efficiency because there is no need to add free PNIPAAm later to improve thermal precipitation efficiency. The study specifically investigated the synthetic conditions based on monomer ratio and amount of introduced CTA because increasing the thermal precipitation efficiency of conjugates improves the recovery of conjugated protein, allowing for a broader range of future applications across various fields. Finally, this study tried to demonstrate the adsorption and removal of uremic toxin, indoxyl sulfate (IS) using human serum albumin (HSA)-PNIPAAm conjugate (Figure 1).

## 2. Results and Discussion

### 2.1. Synthesis of BSA-CTA

**Figure 2:**
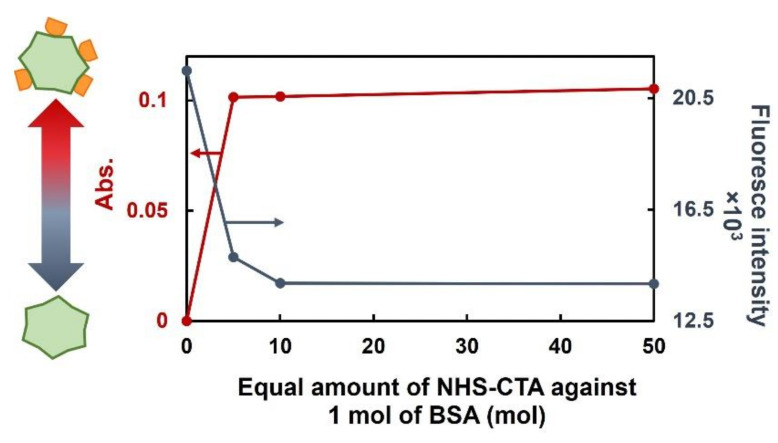
(**Left**) UV absorbance of BSA-CTA with different feeding ratios at 310 nm. The absorbance intensity was calculated by comparing thiocarbonyl group of CTA (310 nm) to BSA (280 nm) (red). (**Right**) Fluorescence intensity of fluorescamine at 495 nm conjugated with unreacted amino residues in BSA-CTA (blue).

First, conjugation of the CTA to BSA was confirmed by UV spectroscopy. Figure 2 (left) shows the absorbance of BSA-CTA at 310 nm which is derived from thiocarbonyl groups (Figure S1). The higher the NHS-CTA ratio, the higher its absorbance. The peak reached a plateau at 5 mole equivalents against BSA. This result is in a good agreement with previous reports that used NHS-CTA for reaction with protein amino groups [24]. The successful conjugation was also confirmed by observing unreacted amino residues in the BSA. The unreacted amino residues were labeled with fluorescamine and its fluorescence intensity was observed. The results that are shown in Figure 2 (right) indicate that by increasing the amount of NHS-CTA, the fluorescence intensity of fluorescamine at 495 nm increased, which indicates that bare amino residues decreased. The intensity reached a plateau around 5–10 mole equivalent against BSA. This shows a quantitative relationship between the introduced CTA group and modified amino residue, suggesting that the amount of CTA introduced can be controlled by feeding ratio of NHS-CTA. Table 1 summarizes the feeding ratios and the obtained intensities.

### 2.2. Synthesis of BSA-PNIPAAm Conjugates

First, the model reaction between CTA and monomer only was carried out in PB and the results showed that the molecular weight distribution was narrower than that of free radical polymerization, indicating that the polymerization could be controlled (Figure S2). Next, the preparation conditions for BSA-PNIPAAm conjugates were optimized by changing monomer concentrations (Figure S3). The successful conjugation was confirmed from SDS-PAGE results by comparing the molecular weight bands. The conjugates prepared with 800 mM showed a band broadening compared to 400 mM, indicating an increase in the molecular weight (Figures S3 and S4). These findings were in parallel with our previous studies that approved the successful polymeric antibody conjugation [22,23]. Moreover, from ^1^H NMR, the change in monomer conversion over time confirmed 100% conversion 48 h after the reaction (Figure S5). Based on these results, the monomer concentration was set at 800 mM and the reaction time at 48 h before the subsequent reaction conditions were decided. Under these conditions, various BSA-PNIPAAm conjugates with different monomer ratios and CTA groups were prepared to investigate the effect of polymer chain length and the number of polymers by comparing the monomer ratio and the amount of CTA, respectively (Table 2).

**Figure 3:**
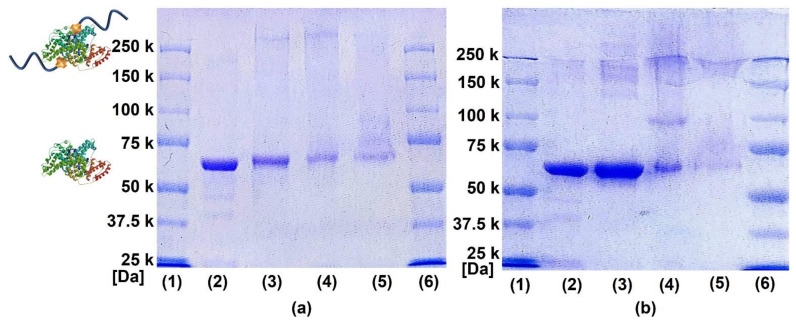
SDS-PAGE analysis for BSA-PNIPAAm conjugates: (**a**) Monomer ratio to CTA of BSA-PNIPAAm conjugates (lanes: (1) protein standard (2) BSA (3) B/C5/PN400 (4) B/C5/PN800 (5) B/C5/PN1600 (6) protein standard). (**b**) CTA ratio to BSA of BSA-PNIPAAm conjugates (lanes: (1) protein standard (2) BSA (3) B/C2/PN800 (4) B/C5/PN800 (5) B/C10/PN800 (6) protein standard).

The SDS-PAGE bands in Figure 3a compare samples with different monomer ratios. The band of native BSA was confirmed around 66 kDa. The higher monomer ratio had shown band broadening at the higher end of molecular weight spectrum more than 66 kDa. This result suggests that an increase in the molecular weight of the conjugates was caused by the increase in the monomer ratio. In contrast, measurement results shown in Figure 3b confirm the band for unreacted BSA becomes thinner with increase in the amount of CTA introduced. This result suggests that CTA introduction amount is directly contributed to improve polymer introduction efficiency. These results were in good agreement with previous papers that characterized and identified a wide range of bands in BSA-PNIPAAm conjugates [24,27].

### 2.3. Thempretaure-Responsive Phase Transition Behavior

**Figure 4:**
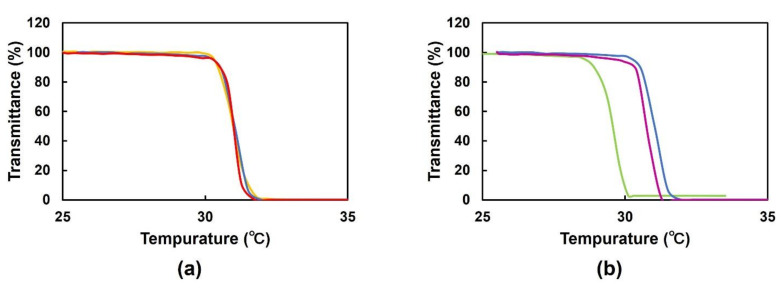
Transmittance of the BSA-PNIPAAm solutions against temperature (thermal-response): (**a**) Monomer ratio to CTA. B/C5/PN400 (yellow), B/C5/PN800 (blue), B/C5/PN1600 (red). (**b**) CTA ratio to BSA. B/C2/PN800 (green), B/C5/PN800 (blue), B/C10/PN800 (purple). (solvent: PBS (pH: 7.4), BSA concentration: 1.0 mg/mL, heating rate: 1 °C/min, wavelength: 450 nm).

The temperature-responsive phase transition before and after the conjugation was observed by measuring the LCSTs. LCST was defined as the temperature at which 50% transmittance was observed. The LCST of the conjugate was observed around 31.0 °C, confirming that the prepared conjugates are temperature responsive (Figure 4a). In general, LCST shifts when PNIPAAm is conjugated to proteins by GT method due to the introduced functional group (binding site) in the polymer side or end chain. Indeed, the LCST of antibody-PNIPAAm conjugates prepared by GT method was 37 °C in our previous study [22,23]. Therefore, it may be one of the advantages for GF method that homo NIPAAm can be polymerized from proteins without any comonomers. It may be also plausible that unconjugated PNIPAAm in the solution can affect the phase transition. Therefore, we have also conducted the solution behavior by dynamic light scattering in the following section.

### 2.4. Temprature-Responsive Aggregation Behavior

**Figure 5:**
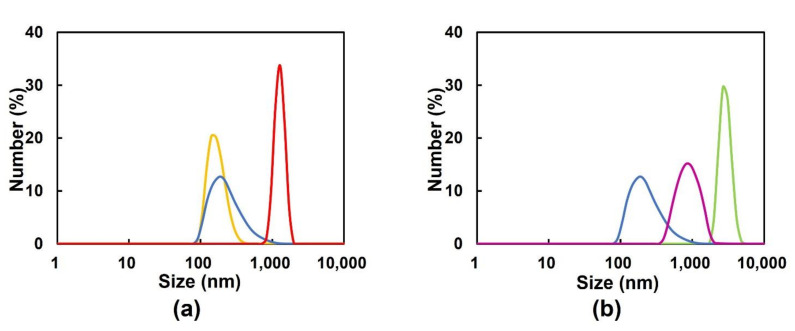
Confirmation of particle size of BSA-PNIPAAm conjugates by DLS at 35 °C. (**a**) Monomer ratio to CTA. B/C5/PN400 (yellow), B/C5/PN800 (blue), B/C5/PN1600 (red). (**b**) CTA ratio to BSA. B/C2/PN800 (green), B/C5/PN800 (blue), B/C10/PN800 (purple).

To investigate the dispersibility and temperature-responsive aggregation behavior for the conjugates, DLS measurement was conducted at 35 °C (above the LCST) (Table 3). The DLS results shown in Figure 5a confirm the formation of a few hundred nm polymer particles in the samples with monomer ratios of 400 and 800 mol%. The formation of polymer particles is caused by the phase transition of PNIPAAm, forming the hydrophobic core surrounded by BSA shell. Other literatures also suggested that protein-PNIPAAm conjugates form stable aggregates above the LCST [14,20,28,30,31]. Of particular interest is that the conjugates with less than 2-mole equivalents of CTA did not form the aggregate plausibly due to the insufficient amount of introduced PNIPAAm. On the other hand, the conjugates with 10-mole equivalents amount of CTA did not form stable, monodisperse aggregate [20]. These results indicate that control of CTA introduction directly affects the phase transition behavior of BSA-PNIPAAm conjugates.

### 2.5. Thermal Precipitation and Recovery of BSA-PNIPAAm

**Figure 6:**
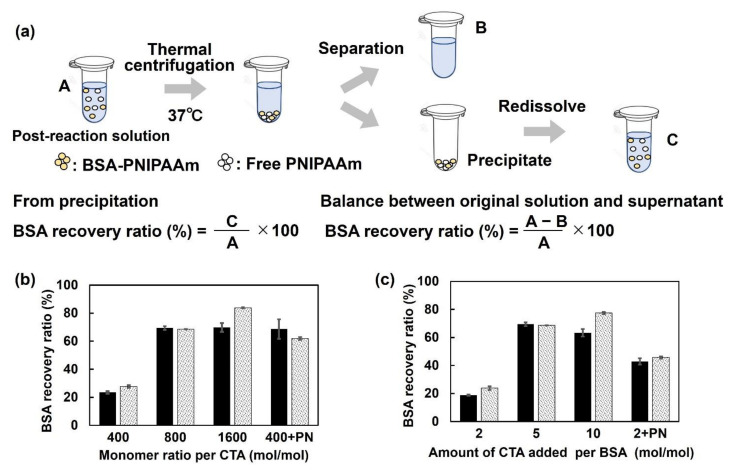
(**a**) The process of BSA-PNIPAAm conjugates thermal precipitate. (**b**) Effects of monomer ratio to CTA on BSA recovery ratio (PN: Free PNIPAAm). (**c**) Effects of CTA ratio to BSA-on BSA recovery ratio (PN: Free PNIPAAm). Calculated from precipitation (black) and a balance between original solution and supernatant (gray). (mean ± SD, *n* = 3).

Figure 6a depicts the thermal precipitation protocol of BSA-PNIPAAm. The BSA-PNIPAAm recovery ratio was calculated from the precipitated BSA after thermal precipitation. About 20% of BSA-PNIPAAm was recovered for the samples with a monomer ratio of 400-mole, while 70% was recovered when 800 and 1600-mole equivalents samples were used (Figure 6b). From this calculation, no difference was observed between the sample with 800 and 1600-mole equivalents even though the 1600 sample should have higher molecular weight and thus precipitate more. This may be caused by weight loss of the sample during precipitation and re-suspending process. In addition, unconjugated free PNIPAAm may affect bicinchoninic acid (BCA) assay. Therefore, to minimize the error, the recovery ratio was also calculated from a balance between the original solution and supernatant solution as shown in Figure 6a. From this calculation, about 80% of BSA-PNIPAAm was recovered when the monomer ratio was 1600-mole equivalents. These results suggest that feeding monomer ratio (that is, the molecular weight of PNIPAAm) influences thermal precipitation efficiency. Indeed, the recovery ratio for the samples with a monomer ratio of 400-mole increased to 60% when free PNIPAAm was added (400 + PN).

Figure 6c shows the effects of CTA on the recovery of BSA-PNIPAAm. The recovery ratios were about 20%, 70% and 80% for the sample with CTA of 2 mole, 5 mole and 10 mole, respectively. Because amount of introduced CTA directly affects the number of polymer chains on BSA, the sample with higher CTA concentration resulted a higher recovery ratio. The obtained recovery ratio was higher than the ones in previously reported systems using the GT method [22,23].

### 2.6. Evaluation of Indoxyl Sulfate (IS) Adsorption Capacity

**Figure 7:**
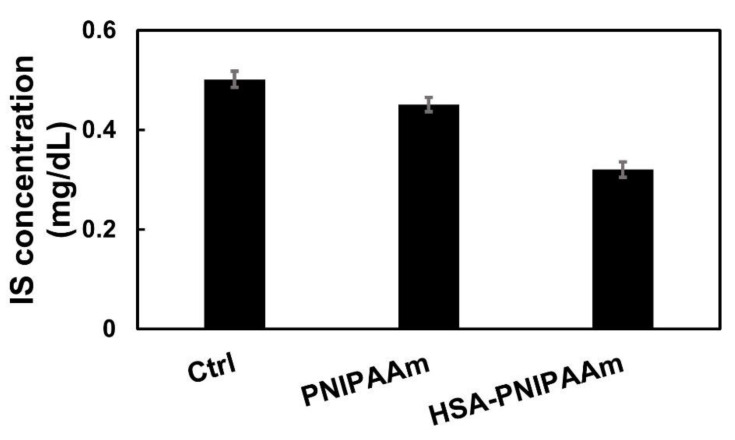
IS concentration in the supernatant after IS adsorption and thermal precipitation using HSA-PNIPAAm conjugate (mean ± SD, *n* = 3).

Finally, we have examined the adsorption and removal ability of our conjugates toward indoxyl sulfate (IS). IS is one of the uremic toxins that cannot be easily removed via hemodialysis. IS has been also known as a protein-bound molecules, especially human serum albumin (HSA) [32]. Therefore, we have newly designed HSA-PNIPAAm conjugates (H/C5/PN800 in Table 2). First, the IS adsorption isotherms of HSA and HSA-PNIPAAm were prepared (Figure S6). The results showed that HSA conjugated with PNIPAAm had the same IS adsorption capacity as average HSA. Next, thermal precipitation was carried out after the IS adsorption and the concentration of IS in the supernatant was measured, as shown in Figure 7. The HSA-PNIPAAm conjugates reduced the IS concentration by about 40% compared to the other conditions, indicating the possibility of IS removal by simple thermal precipitation. These results also suggest that PNIPAAm can be introduced into albumin without impairing its activity. Previously, the combination of polymers and adsorbents to remove urinary toxins by different methods have been investigated and reported [33,34]. In the current approach, the HSA-PNIPAAm conjugates showed IS adsorption ability that is expected to be applied to chronic kidney disease (CKD) and cardiovascular disease (CVD) treatment [35,36]. In particular, in the molecular adsorbent recirculating system (MARS) which uses albumin dialysis, if albumin can be simply recovered from the dialysate, it will reduce medical costs. In addition, the introduction of PNIPAAm into proteins other than BSA (e.g., antibodies) using the same technique will enable the creation of conjugates more quickly than the conventional method. This will facilitate the development of different medical approaches, including diagnostics.

## 3. Materials and Methods

### 3.1. Materials

*N*-Isopropylacrylamide (NIPAAm, Fujifilm Wako Pure Chemical (Osaka, Japan), 97%) was recrystallized from hexane and dried under vacuum before use. 2,2′-Azobis [2-(2-imidazolin-2-yl) propane] Dihydrochloride (VA-044, Tokyo Kasei, 98.0%) was recrystallized from methanol and dried under vacuum before use. 2-(Dodecylthiocarbonothioylthio)-2-methylpropionic acid *N*-hydroxysuccinimide ester (NHS-CTA, Sigma-Aldrich, St. Louis, MO, USA), *N*,*N*-Dimethylformamide (DMF, 99.5%, Fujifilm Wako Pure Chemical), 0.1 mol/l-phosphate buffer solution (PB, Nacalai Tesque, Kyoto, Japan), Dulbecco’s phosphate-buffered saline (PBS, Sigma-Aldrich), fluorescamine (Tokyo Kasei), Methanol (99.8%, Fujifilm Wako Pure Chemical), 10×Tris/Glycine/SDS buffer (BIO-RAD), Coomassie brilliant blue R-250 (CBB, BIO-RAD), Laemmli sample buffer (BIO-RAD), 2-Mercaptoethanol (Fujifilm Wako Pure Chemical, 99%), Precision plus protein unstained standards (BIO-RAD), Bovine serum albumin (BSA, 66 kDa, Sigma-Aldrich) Human Serum Albumin (HSA, Nacalai Tesque), Micro BCA™ Protein Assay Kit (Thermo Fisher Scientific, Waltham, MA, USA), 3-Indoxyl sulfate potassium salt (Carbosynth, Compton, UK), IS assay kits (NIPRO, Osaka, Japan).

### 3.2. Synthesis of BSA-CTA

BSA (66.4 mg, 7 μmol) and 55 mL phosphate buffer (PB) (0.1 M, pH 8.0) were added to 100 mL eggplant flask with a 3-way stopcock and dissolved for 30 min under argon atmosphere. Next, a mixture of NHS-CTA (16.3 mg, 35 μmol) dissolved in 3 mL of DMF was added dropwise to the previous solution and the reaction was continued for 24 h in a 25 °C water bath. The reaction mixture was dissolved in 55 mL of PB (0.1 M, pH 8.0) for 15 min and the unwanted material was removed by filter paper (4 μm) and dialysis (MWCO: 12,000–14,000) in 1 L, three times (5 h, 12 h, 24 h) with distilled water exchange. The white powder was then recovered by lyophilization.

The amount of CTA group introduced was evaluated by measuring the absorbance intensity of the thiocarbonyl group-derived 310 nm by UV-vis. Amino residues were also measured by fluorescamine. BSA-CTA (0.5 mg/mL, 50 µL) and BSA (0.5 mg/mL, 50 µL) were added to 96 well plates and fluorescamine dissolved in acetone (50 mg/mL, 5 µL) was added to each well. The fluorescence of each sample was measured using the Infinite M Nano (TECAN, Männedorf, Switzerland) at an excitation wavelength of 395 nm and a measurement wavelength of 495 nm.

### 3.3. BSA-PNIPAAm Conjugates Adjustment Conditions Investigation

The synthesized BSA-CTA (66 mg, 1 μmol) and 4 mL of PB (0.1 M, pH 6.0) were added to a 10 mL sample tube and dissolved for 30 min. Then, NIPAAm (452.6 mg, 4 mmol) was added and dissolved in the mixed solution and 1 mL of PB containing (50 μmol, 16 mg) of initiator VA-044 was added dropwise. Then, the reaction was sealed with argon and carried out in a water bath at 30 °C for 48 h.

### 3.4. BSA-PNIPAAm Conjugates Monomer Ratios and Introduced CTA Groups Investigation

To investigate monomer ratios, NIPAAm was added in equal amounts of 400 mole, 800 mole and 1600 mole to the CTA groups introduced into BSA. The conjugates were prepared under each condition according to the previous protocol described in (Section 3.2). To investigate the amount of CTA groups introduced into BSA, NHS-CTA was added in equal amounts of 2 mosle, 5 mole and 10 mole against BSA to synthesize BSA-CTA for each condition. Each preparation of the conjugates used the BSA-CTA. BSA-PNIPAAm conjugates properties were evaluated using SDS-PAGE, UV-vis and DLS.

### 3.5. SDS-PAGE Measurement

The succeeded conjugates were characterized by sodium dodecyl sulfate-polyacrylamide gel electrophoresis (SDS-PAGE) compared with BSA and polymer, each one in isolation. The gray scale of SDS-PAGE of each sample was measured by ImageJ (ver. 1.52a, National institutes of health, Bethesda, MD, USA).

### 3.6. Lower Critical Solution Temperature Measurement

The temperature dependence of the transmittance in the prepared samples was measured using a spectrophotometer. The prepared conjugates solution was diluted and adjusted to 1 mg/mL BSA concentration with PBS. The sample solution and the stirring bar were subjected to absorbance measurement using a spectrophotometer in a nitrogen atmosphere at a wavelength of 450 nm, a temperature range of 25 °C to 40 °C and a temperature rising rate of 1.0 °C/min were applied for measurements [22].

### 3.7. DLS Measurement

The prepared conjugate solution was diluted and adjusted with PBS to the BSA concentration of 1 mg/mL and measured using a Malvern Zetasizer-Nano ZSP at λ = 633 nm, scattering angle 173° and 35 °C. The diameter and aggregation state of samples were evaluated.

### 3.8. BSA Recovery Ratio Evaluation

The prepared conjugate solution was diluted with PBS to a BSA concentration of 1 mg/mL and 1 mL was added to a 2 mL microcentrifuge tube and centrifuged at 37 °C, 15,000 rpm for 15 min. After aliquoting 900 μL of the supernatant, 900 μL of PBS was added and the solution was re-dissolved. The BSA concentration of the solution was then measured by the BCA method and the recovery ratio was calculated.

### 3.9. The BCA Method

The prepared conjugate solution was diluted with PBS and adjusted 0.25 mg/mL BSA concentration. Then, 25 μL of this solution and 200 μL of working solution (Micro BCA™ Protein Assay Kit) were added to 96 well plates and incubated at 37 °C for 30 min. The absorbance of each sample was measured at a wavelength of 562 nm and the BSA concentration was calculated based on the calibration curve of BSA (0, 0.25, 0.5, 1.0, 2.0 mg/mL).

### 3.10. Indoxyl Sulfate (IS) Adsorption Capacity Evaluation

HSA-PNIPAAm conjugates and IS were dissolved in PBS with 1.0 mg/mL HSA-PNIPAAm constant concentration and IS different concentrations 0–160 µg/mL. The solution was shaken at 25 °C for 2 h and then centrifuged at 10,000 rpm for 10 min using a 10k centrifuge membrane. The IS adsorption capacity of the HSA-PNIPAAm conjugates were prepared and calculated by measuring the IS concentration of the supernatant using the IS measuring reagent “NIPRO” and the adsorption isotherms were plotted out.

For thermal precipitation evaluation, the solution with HSA-PNIPAAm concentration of 2.0 mg/mL and IS concentration of 5.4 µg/mL was prepared with PBS, followed by shaking at 25 °C for 2 h. After thermal precipitation by centrifugation at 37 °C, 15,000 rpm for 15 min, the IS concentration of the supernatant was measured.

### 3.11. GPC and NMR Measurements

Gel permeation chromatography (GPC) and a spectrophotometer. In ^1^H NMR, D_2_O was used as a solvent. For GPC (Solvent: THF, Standard: poly (styrene)), the number average molecular weight (Mn), weight average molecular weight (Mw) and molecular weight distribution (Mw/Mn) of the prepared samples were measured.

## 4. Conclusions

This study successfully synthesized BSA-PNIPAAm conjugates using the GF strategy. The BSA-PNIPAAm conjugates showed different properties by changing the monomer ratio and introducing CTA at low monomer ratios. The high monomer ratio shows high thermal precipitation efficiency. The results indicated that the number of PNIPAAm introduced and a certain amount of chain length affected the thermal precipitation efficiency of the BSA-PNIPAAm conjugates. In addition, HSA-PNIPAAm conjugates showed the ability to adsorb IS, indicating that the activity of albumin was retained during conjugation process. To the best of our knowledge, this research is the first to enhance protein introduction to responsive polymers for uremic toxins purification. The proposed technology is efficient for introducing polymers into proteins, providing wider range of applications in the medical field than traditional methods. In addition, turning albumin into other proteins, such as antibodies, can be used for drug delivery systems (DDS) and diagnostic approaches, especially during the current SARS-CoV-2 pandemic situations.

## Figures and Tables

**Table 1:** Feeding ratios of BSA-CTA.

Sample Name	BSA (μmol)	NHS-CTA (μmol)	Abs. (310 nm/280 nm) *	Fluorescence Intensity ×10^3^ (495 nm) **
BSA-CTA 0	1	0	0	21.5
BSA-CTA 5	1	5	0.1014	14.8
BSA-CTA 10	1	10	0.1017	13.9
BSA-CTA 50	1	50	0.1052	13.8

* The absorbance intensity derived from thiocarbonyl group (310 nm) against absorbance derived from protein (280 nm). ** The fluorescence intensity derived from fluorescamine (495 nm) conjugated to unreacted amino residues.

**Table 2:** Feeding ratios of BSA-PNIPAAm.

Sample Name	Molar Ratio	
	CTA/BSA	Monomer/CTA
BSA-CTA5-PNIPAAm400 (B/C5/PN400)	5	400
BSA-CTA5-PNIPAAm800 (B/C5/PN800)	5	800
BSA-CTA5-PNIPAAm1600 (B/C5/PN1600)	5	1600
BSA-CTA2-PNIPAAm800 (B/C2/PN800)	2	800
BSA-CTA10-PNIPAAm800 (B/C10/PN800)	10	800
HSA-CTA5-PNIPAAm800 (H/C5/PN800)	5	800

**Table 3:** DLS results of each sample.

Sample Name	Size (nm)	PdI
BSA-CTA5-PNIPAAm400 (B/C5/PN400)	170	0.04
BSA-CTA5-PNIPAAm800 (B/C5/PN800)	250	0.20
BSA-CTA5-PNIPAAm1600 (B/C5/PN1600)	1280	0.04
BSA-CTA2-PNIPAAm800 (B/C2/PN800)	3000	0.13
BSA-CTA10-PNIPAAm800 (B/C10/PN800)	920	0.19

## Data Availability

The data that support the findings of this study are available from the corresponding author, M.E., upon reasonable request.

## Supplementary Materials

The following supporting information can be downloaded online. Table S1: Feeding ratio of BSA-PNIPAAm for the investigation of monomer concentration; Figure S1: UV-vis measurement results of each BSA-CTA in water (1 mg/mL); Figure S2: The GPC results of PNIPAAm with and without CTA; Figure S3: Monomer concentration of BSA-PNIPAAm conjugates observed through SDS-PAGE. (Red arrows, no band broadening while yellow arrows show band broadening after conjugation); Figure S4: Gray value of the SDS-PAGE result calculated by image J. (The band on the high molecular weight side with 800 mM was more deep color); Figure S5: ^1^H NMR spectrum of BSA-PNIPAAm in D_2_O; Figure S6: Adsorption isotherm of each sample.

## Sample Availability

Samples of the compounds are not available from the authors.
