# Supplementary material for: Temperature Responsive Polymer Conjugate Prepared by “Grafting from” Proteins toward the Adsorption and Removal of Uremic Toxin
Source: Molecules. 2022 Feb 3;27(3):1051. doi: 10.3390/molecules27031051 (PMC8839407; doi:10.3390/molecules27031051)
Supplement: Supplementary file 1 [file molecules-27-01051-s001.zip › molecules-1568296-supplementary.pdf]

Supporting information

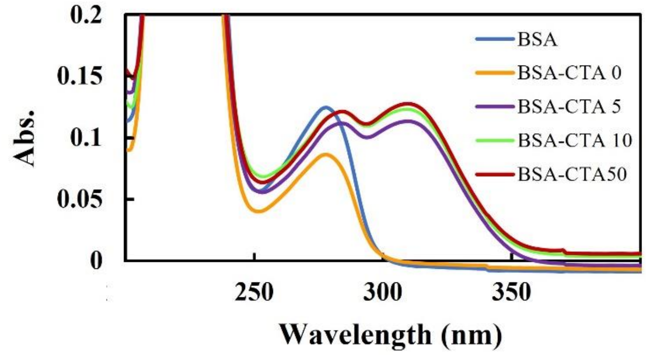

Figure S1. UV-vis measurement results of each BSA-CTA in water (1 mg/mL).

Table S1. Feeding ratio of BSA-PNIPAAm for the investigation of monomer concentration

Sample name	BSA [μmol]	NIPAAm [μmol]	VA-044 [μmol]	CTA [μmol]	C [mM]
BSA-CTA5-PNIPAm A	1	400	50	5	800
BSA-CTA5-PNIPAm B	1	400	50	5	400

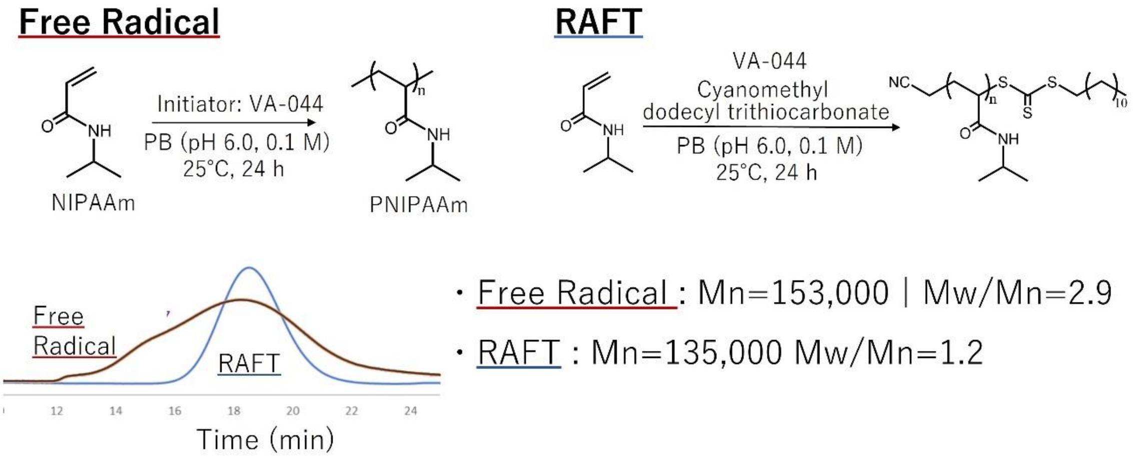

Figure S2. The GPC results of PNIPAAm with and without CTA.

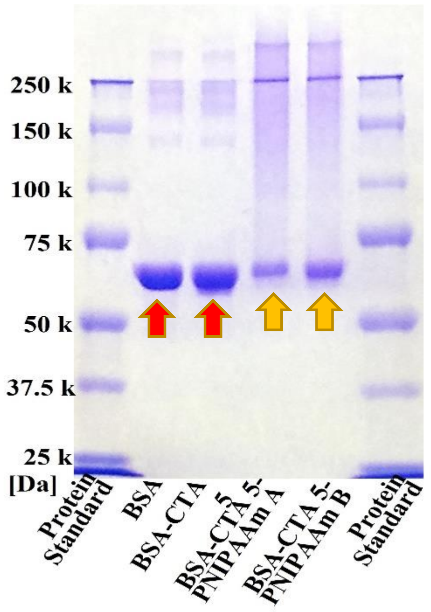

Figure S3. Monomer concentration of BSA-PNIPAAm conjugates observed through SDS-PAGE. (Red arrows, no band broadening while yellow arrows show band broadening after conjugation).

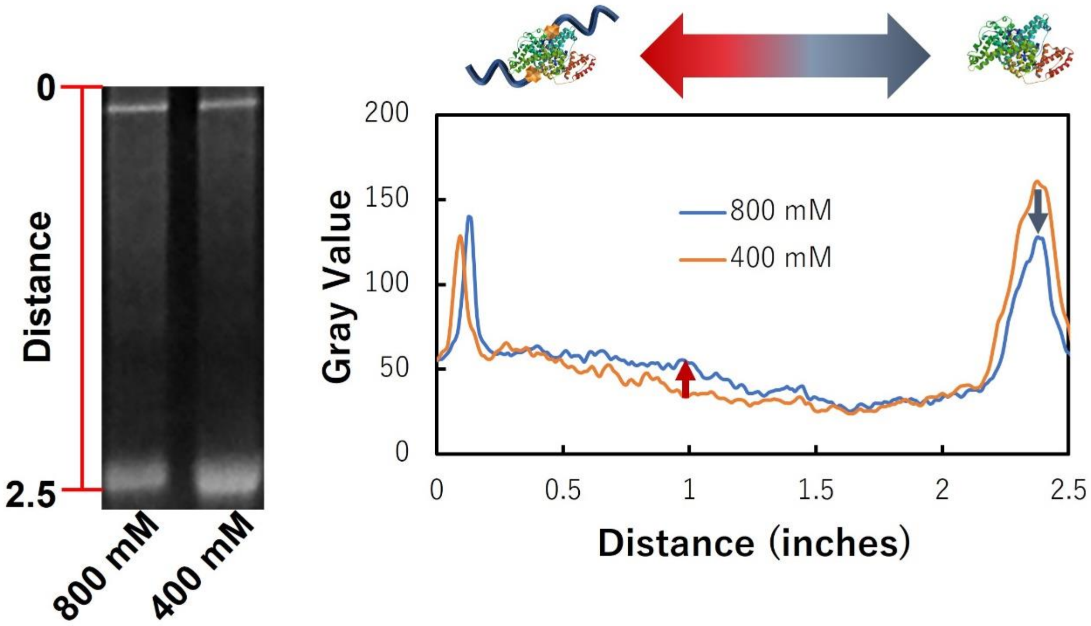

Figure S4. Gray value of the SDS-PAGE result calculated by image J. (The band on the high molecular weight side with 800 mM was more deep color).

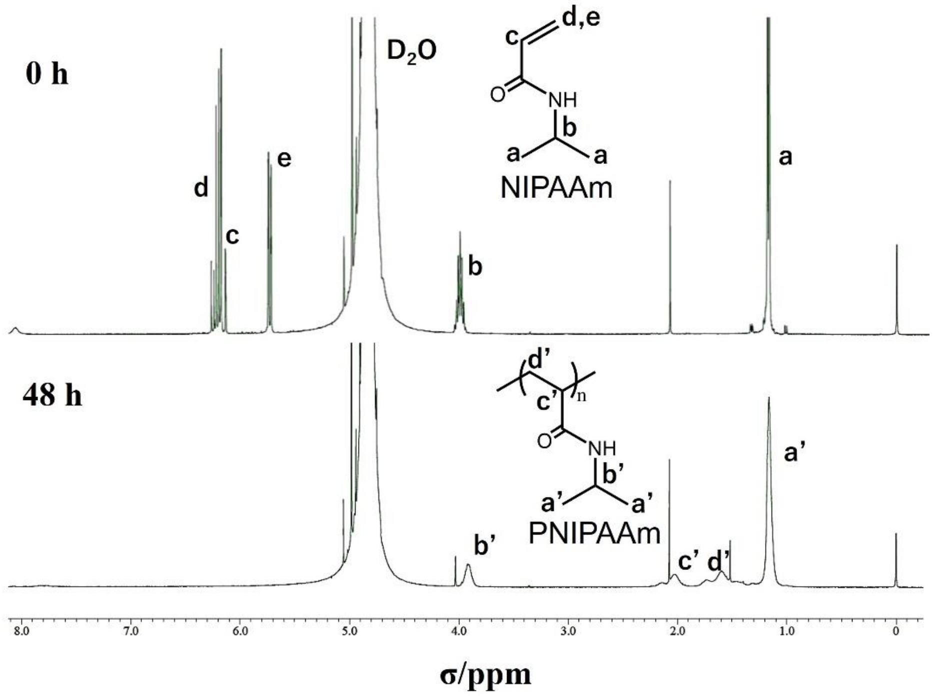

Figure S5. ^{1}H NMR spectrum of BSA-PNIPAAm in D_2O.

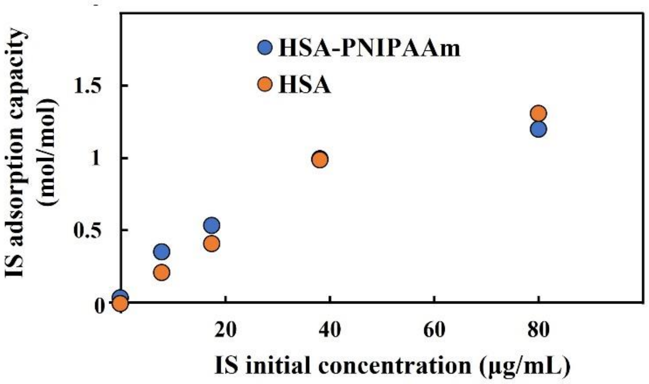

Figure S6. Adsorption isotherm of each sample.
